# Dai-Zong-Fang, A Traditional Chinese Herbal Formula, Ameliorates Insulin Resistance in *db/db* Mice

**DOI:** 10.3389/fphys.2018.00224

**Published:** 2018-03-14

**Authors:** Lili Zhu, Xiaoyun Zhu, Guibo Sun, Xiangbao Meng, Min Wang, Hanming Cui, Jialong Wang, Yadong Zhai, Ke Yang, Yang Tang, Xiaobo Sun, Ximing Liu

**Affiliations:** ^1^Graduate School, Beijing University of Chinese Medicine, Beijing, China; ^2^Guang'anmen Hospital, China Academy of Chinese Medical Sciences, Beijing, China; ^3^Institute of Medicinal Plant Development, Chinese Academy of Medical Sciences & Peking Union Medical College, Beijing, China; ^4^Collaborative Innovation Center of Yangtze River Delta Region Green Pharmaceuticals, Zhejiang University of Technology, Hangzhou, China

**Keywords:** traditional Chinese medicine, Dai-Zong-Fang formula, insulin resistance, hepatic steatosis, skeletal muscle mitochondria, AMPK, notch signaling, *db/db* mice

## Abstract

Intricate health problems, such as insulin resistance (IR) and its associated diseases, call for multi-targeted therapies with few side effects. Based on traditional Chinese medicine (TCM), Dai-Zong-Fang (DZF) is an herbal formula mainly composed of Rhizoma Coptidis (Huanglian) and Fructus Aurantii Immaturus (Zhishi), of which berberine and naringin are the main constituents. Though DZF has been clinically used for treatment of IR and metabolic syndrome for decades, its mechanism *in vivo* remains unknown. In the present study, we observed that both DZF and metformin, the first-line drug for type 2 diabetes, ameliorated insulin resistance with significant improvement of oral glucose tolerance test (OGTT) and homeostasis model assessment of IR (HOMA-IR) level in diabetic C57BL/Ksj-Lepr *db*^−/−^ (*db/db*) mice. Low-density lipoprotein cholesterol (LDL-C) and fatty acids (FAs) also decreased in the blood. Higher dose of DZF (1 g·kg^−1^), but not metformin (0.25 g·kg^−1^), alleviated hepatic steatosis with reduced liver weight and hepatic lipid accumulation and provided protection from hepatic injury with lower alanine aminotransferase and aspartate aminotransferase and increased hepatic superoxide dismutase activity in *db/db* mice. Quantitative reverse transcription polymerase chain reaction (qRT-PCR) showed a decrease in FA synthase gene (*Fasn*) and an increase in FA oxidation gene *Ppara* expression. Western blot demonstrated that both DZF and metformin activated 5′ AMP-activated protein kinase (AMPK) but inhibited Notch intracellular domain (NICD) and Hairy/enhancer-of-split 1 (Hes1) of Notch signaling pathway in the liver. DZF also dramatically improved the ultrastructure of skeletal muscles, AMPK phosphorylation, and GLUT4 translocation. DZF also promoted FA transport and oxidation with *Cd36* and *Cpt1b* up-regulation in the skeletal muscle. In conclusion, DZF improves insulin sensitivity by reducing hepatic lipids through AMPK activation and Notch signal pathway inhibition and enhancing energy metabolism in the skeletal muscle via AMPK. This study provides insights into the treatment of complex conditions, such as IR, where TCM herbal formulas exert multipronged effects through correlating pathways.

## Introduction

With the epidemic of obesity, rising prevalence is observed in intertwined diseases, such as metabolic syndrome (MetS), nonalcoholic fatty liver disease (NAFLD), type 2 diabetes mellitus (T2DM), and atherosclerotic heart disease (Samuel and Shulman, 2012). T2DM is one of the most urgent health problems, affecting 425 million adults across the globe and leading to substantial complications (IDF, 2017). Insulin resistance (IR) is a forerunner and hallmark of T2DM and also a common pathophysiological mechanism of MetS and NAFLD, for which therapies mainly rely on lifestyle and weight loss (Asrih and Jornayvaz, 2015). IR is associated with myocardial dysfunction before the onset of diabetes (Fontes-Carvalho et al., 2015). Thus, understanding IR pathogenesis and finding proper therapies have become increasingly important.

Systemic IR results from the interactions of different tissues and cell types (Tomas et al., 2004; Samuel and Shulman, 2012; Odegaard and Chawla, 2013). Accumulation of ectopic lipids in the liver, muscle, or other tissues, together with mitochondrial defects, converge to promote IR (Samuel and Shulman, 2016). The liver and skeletal muscles both serve as depots of energy and are also main tissues of lipid and glucose metabolism. Hepatic lipid deposits contribute to fasting blood glucose (FBG), dyslipidemia, and IR and probably form prior to alterations in other tissues (Kotronen et al., 2008). Skeletal muscle plays an important role in peripheral glucose uptake, insulin-stimulated glucose disposal, and whole-body energy homeostasis (Shulman et al., 1990). Fatty acids (FAs) partly originate from the hydrolysis of triglyceride (TG)-rich lipoproteins, which are secreted by the hepatic tissue, and can be uptaken by skeletal muscle mitochondria for oxidation (Goldberg and Ginsberg, 2006). Skeletal muscle mitochondria provide a platform for ATP generation by oxidation of lipids and carbohydrates (Montgomery and Turner, 2015), and its dysfunction (possibly due to excessive FA influx) serves as an important factor in IR pathogenesis (Montgomery and Turner, 2015; Turner and Robker, 2015), which promotes energy conversion into hepatic *de novo* lipogenesis, thus promoting atherogenic dyslipidemia (Kotronen et al., 2008).

IR occurs under any defects of the insulin signaling pathway. Insulin binds to the subunit of insulin receptor, phosphorylates the insulin receptor substrate (IRS) proteins, and then promotes activation of phosphatidylinositol 3 kinase (PI3K) and protein kinase B (PKB [Akt]), which are essential for reduction of hepatic glyconeogenesis and insulin-stimulated translocation of glucose transporter 4 (GLUT4) to the plasma membrane in the skeletal muscles (Okada et al., 1994). In recent years, studies have revealed the key role of Notch signaling pathway, which is regarded a novel regulator of metabolism (Bi and Kuang, 2015). Abnormal activation of Notch signaling in the liver is associated with hepatic lipogenesis, thus inducing IR, hyperglycemia, and fatty liver disease (Pajvani et al., 2011, 2013; Valenti et al., 2013; Geisler and Strazzabosco, 2015); however, Notch signaling can be inhibited by 5′ AMP-activated protein kinase (AMPK) activation (Li et al., 2014). AMPK is a fuel-sensing enzyme, and it can regulate a wide array of events, such as glucose transport, FA oxidation, and mitochondrial function. (Ruderman et al., 2013). Pharmacological AMPK activation (Ruderman et al., 2013) and Notch inhibition (Liu et al., 2013) improve IR.

The concept of wholism is a major characteristic of traditional Chinese medicine (TCM). In the perception of intricate problems, such as IR, TCM provides insights from a macroscopic perspective. Dai-Zong-Fang (DZF), an herbal formula based on TCM, has been used in TCM clinic for decades and mainly composed of Rhizoma Coptidis (Huanglian) and Fructus Aurantii Immaturus (Zhishi). In previous studies, DZF was proven to be significantly effective in glucose and lipid disorders in patients with metabolic syndrome (Zhu et al., 2017). DZF lowered blood glucose and serum lipids and ameliorated IR in diabetic KKAy mice (Zhao, 2017). In *in vitro* studies, DZF inhibited intracellular lipid accumulation in HepG2 cells induced by oleic acid (Dong et al., 2015). DZF also promotes glucose consumption and regulates the dynamic balance of lipolysis and lipogenesis in 3T3-L1 adipocytes (Zhu et al., 2015). DZF increases glucose uptake in C2C12 skeletal muscle cells by promoting GLUT4 translocation (Zhao et al., 2016). However, no study has elucidated the *in vivo* mechanism, which is essential due to systemic interaction, for the effect of DZF. In the present study, we further defined the effects of DZF on insulin-resistant (Kodama et al., 1994), obese, and diabetic C57BL/Ksj-Lepr *db*^−/−^ (*db/db*) mice and the underlying mechanisms.

## Results

### Qualitative analysis of bioactive compounds in DZF formula

**Figure 1:**
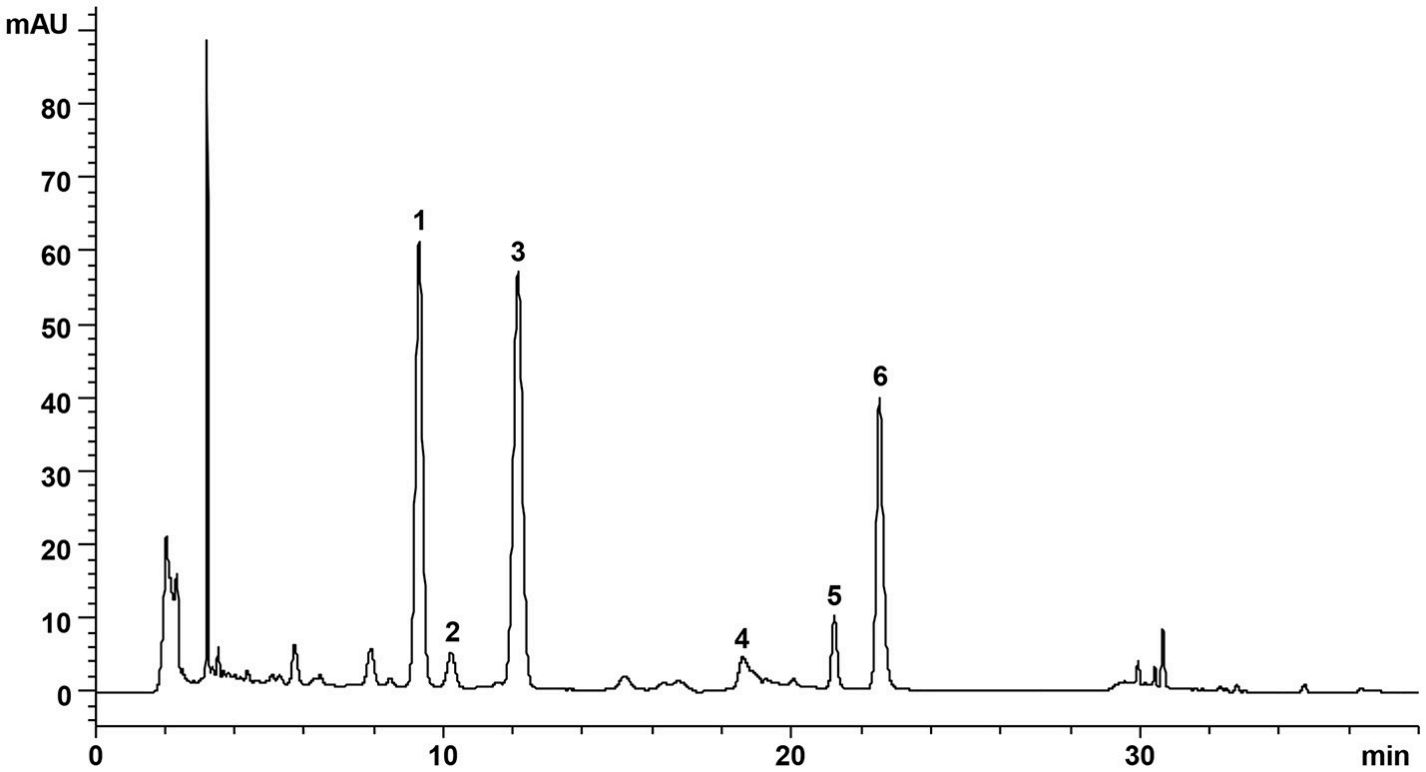
Representative chromatogram of major compounds in DZF. 1. Naringin; 2. Hesperidin; 3. Neohesperidin; 4. Jatrorrhizine; 5. Palmatine; 6. Berberine.

DZF extract (dried powder) was provided and produced by Zhejiang Jiu Xu pharmaceutical Co., LTD (Jinhua, China, #20141201). The formula was traditionally prepared as decoction, but it was optimized for better and stable extraction ratio of effective constituents and for improved pharmacological efficacy. The contents of representative chemical compositions in DZF were determined by high-performance liquid chromatography (HPLC), and their constancy and reproducibility were ensured. Figure 1 shows the chromatograms of the primarily identified and quantified components of DZF, and Table 1 lists their specific contents.

**Table 1:** Contents of the main identified compounds in DZF extract.

**Herbs**	**Compounds**	**Contents (mg/g)**
Rhizoma Coptidis (Huanglian)	Berberine	28.90
	Palmatine	5.50
	Jatrorrhizine	3.93
Fructus Aurantii Immaturus (Zhishi)	Naringin	73.90
	Hesperidin	8.61
	Neohesperidin	87.09

### Fasting blood glucose and body weight of *db/db* mice

**Figure 2:**
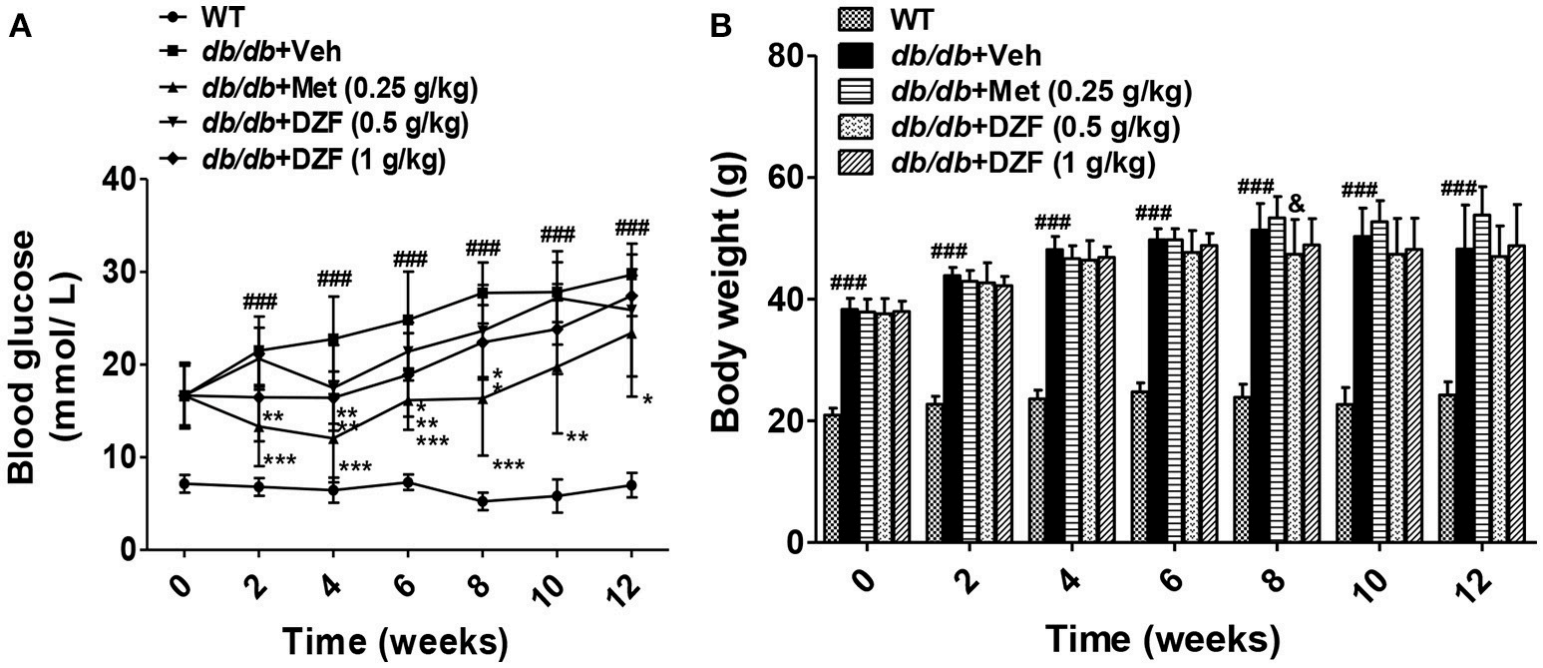
Effects on FBG **(A)**, body weight **(B)** after vehicle, metformin, and DZF administration in *db/db* mice. Data are described as mean ± SD (*n* = 10). ^###^*p* < 0.001 vs. WT control; ^*^p < 0.05, ^**^p < 0.01, and ^***^p < 0.001 vs. *db/db*+ vehicle. ^&^*p* < 0.05 vs. *db/db*+ metformin.

FBG and body weight were evaluated after 6 h of food removal. Blood was drawn from the tail veins. As shown in Figure 2A, during 12 weeks of observation, *db/db* mice presented significant hyperglycemia compared with the wild-type controls (*p* < 0.001). FBG of the vehicle *db/db* mice continuously increased, whereas metformin administration significantly lowered blood glucose (*p* < 0.05). DZF exerted a hypoglycemic effect on *db/db* mice in the first 8 weeks (*p* < 0.05). With the progress of diabetic conditions, hypoglycemic effect of DZF weakened (*p* > 0.05). No significant difference was observed in body weight between the groups at the end of the experiments. However, metformin presented a weight-gain effect in *db/db* mice (*p* = 0.064), which contradicts clinical observations. At the eighth week, higher body weight was observed in metformin-treated mice than that those administered with DZF (0.5 g·kg^−1^) (*p* < 0.05, Figure 2B).

### DZF improves insulin sensitivity in *db/db* mice

**Figure 3:**
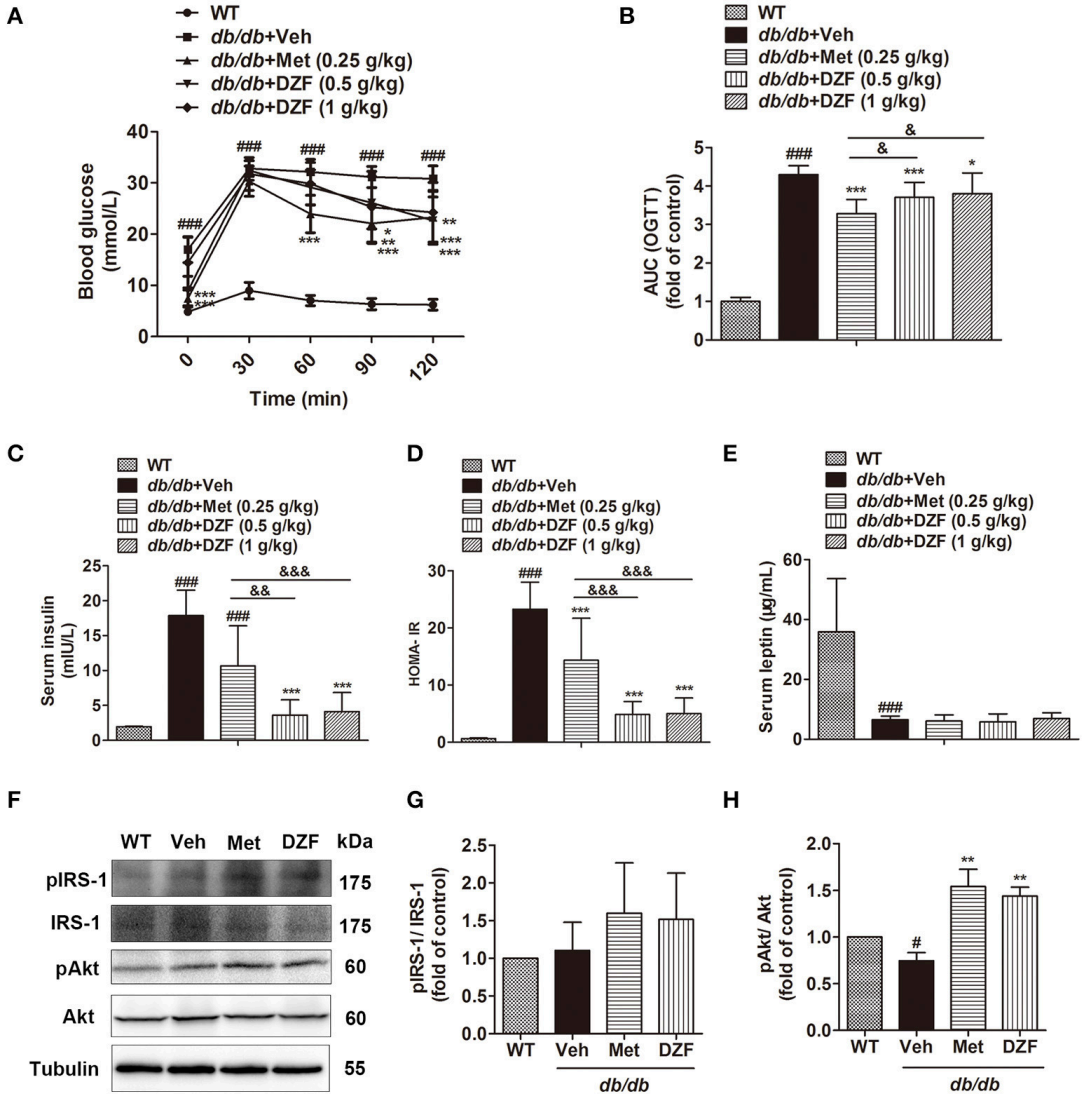
Effects on IR after vehicle, metformin, and DZF administration in *db/db* mice. **(A)** OGTT; **(B)** AUCs of OGTT, normalized by wild-type control; **(C)** Serum insulin concentration; **(D)** HOMA-IR = [fasting glucose (mmol/l)] × [fasting insulin (μU/ml)] /22.5. **(E)** Serum leptin concentration; **(F–H)** Representative immunoblots of IRS-1 and Akt phosphorylation in the liver of wild-type and vehicle-, metformin-, and DZF- (1 g·kg^−1^) treated *db/db* mice. Data are described as mean ± SD. **(A–D)** *n* = 10; **(E)** *n* = 7; **(F–H)** *n* = 3. ^#^*p* < 0.05, ^###^*p* < 0.001 vs. WT control; ^*^p < 0.05, ^**^p < 0.01 and ^***^p < 0.001 vs. *db/db*+ vehicle; ^&^*p* < 0.05, ^&&^*p* < 0.01 and ^&&&^*p* < 0.001 vs. *db/db*+metformin.

Oral glucose tolerance test (OGTT), fasting serum insulin, and homeostasis model assessment of insulin resistance (HOMA-IR) were assessed to determine insulin sensitivity. OGTT was conducted on the 11th week, whereas insulin and HOMA-IR were assessed after 12 weeks of administration. Compared with the wild-type controls, the vehicle *db/db* mice presented significantly higher blood glucose both before and after glucose administration (Figure 3A). Metformin and both doses of DZF reduced the area under the curve (AUC, Figure 3B), indicating that these compounds improved glucose tolerance in *db/db* mice. Fasting serum insulin and HOMA-IR in vehicle *db/db* mice markedly increased compared with wild type controls (*p* < 0.001). Serum insulin level and HOMA-IR significantly reduced after administration of both doses of DZF (*p* < 0.001) and were lower than those with metformin treatment (*p* < 0.01, Figures 3C,D). Leptin levels decreased compared with the wild-type controls (*p* < 0.001) in *db/db* mice. Between the groups of *db/db* mice, there were no significant difference (*p* > 0.05) (Figure 3E). For the investigation of the mechanism of DZF effect, we mainly focused on the higher dose (1 g·kg^−1^) of DZF given that it achieved more manifested effects than the lower measurement (0.5 g·kg^−1^). As shown by Western blot, both DZF (1 g·kg^−1^) and metformin can increase Akt phosphorylation (*p* < 0.01) and phospho-IRS-1 relative expression (Figures 3F–H), indicating that DZF exhibited the potential to stimulate insulin signaling pathway in the liver of *db/db* mice.

### DZF lowers serum low-density lipoprotein cholesterol (LDL-C) and non-esterified FA (NEFA) in *db/db* mice

**Figure 4:**
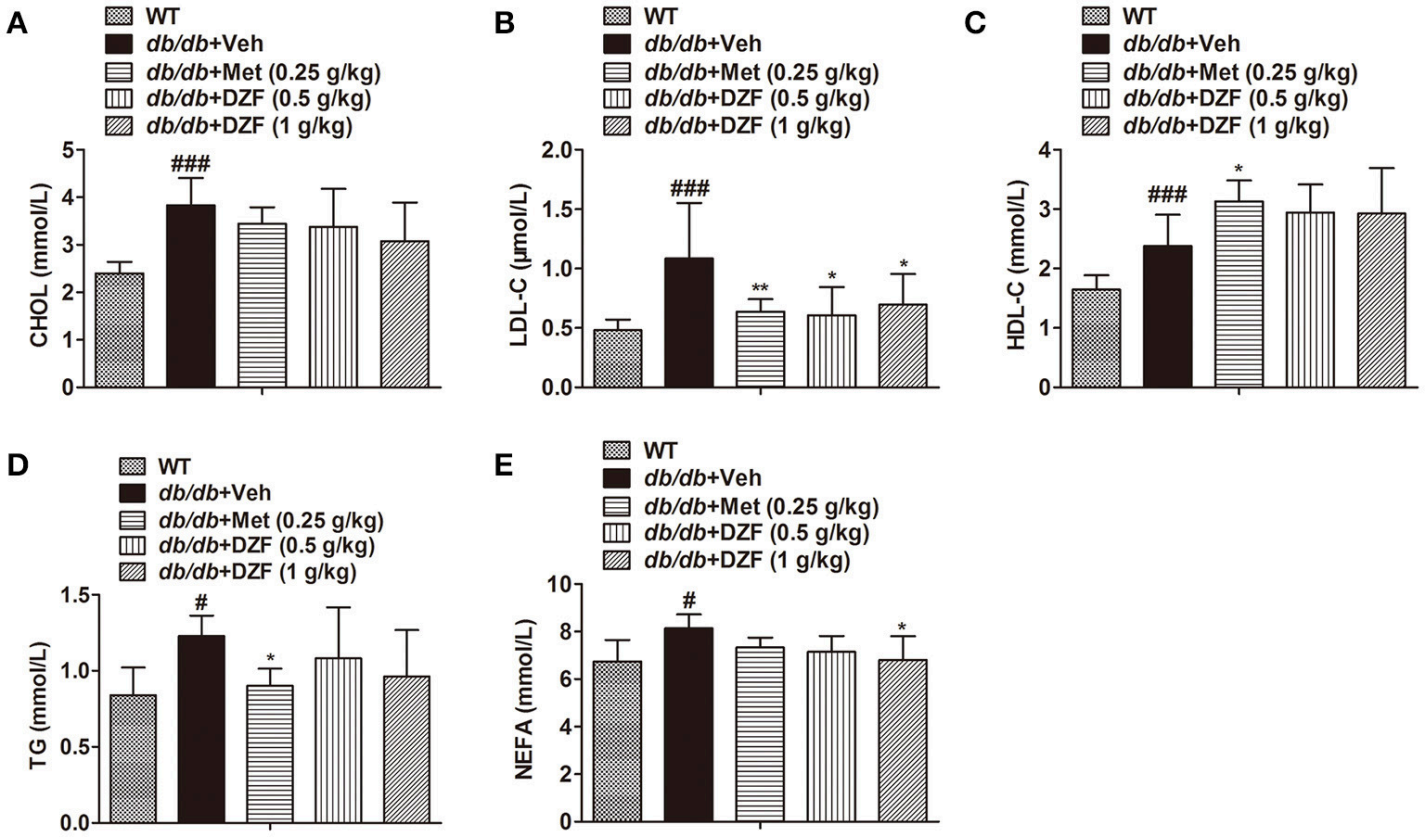
Serum lipid profiles in wild-type and vehicle-, metformin-, and DZF-treated *db/db* mice. **(A)** Serum CHOL level; **(B)** Serum LDL-C level; **(C)** HDL-C level; **(D)** Serum TG level; **(E)** Serum NEFA level. Data are described as mean ± SD. **(A–D)** *n* = 10; **(E)** *n* = 7. ^#^*p* < 0.05, ^###^*p* < 0.001 vs. WT control; ^*^p < 0.05, ^**^p < 0.01 vs. *db/db*+ vehicle.

Serum lipid profiles, including total cholesterol (CHOL), LDL-C, high-density lipoprotein cholesterol (HDL-C), TG, and NEFA, were detected (Figure 4). Compared with the wild-type controls, all lipid profiles presented a notable increase in *db/db* mice (*p* < 0.001 or *p* < 0.05). Metformin significantly reduced LDL-C and TG levels (*p* < 0.01 and *p* < 0.05, respectively) and increased HDL-C level (*p* < 0.05). Both doses of DZF lowered LDL-C, whereas the higher-dose DZF (1 g·kg^−1^) also reduced serum NEFA (*p* < 0.05). In the present study, no significant change was observed in serum CHOL, HDL-C, and TG with DZF administration (*p* > 0.05).

### DZF improves hepatic steatosis and injury in *db/db* mice

#### Hepatic steatosis

**Figure 5:**
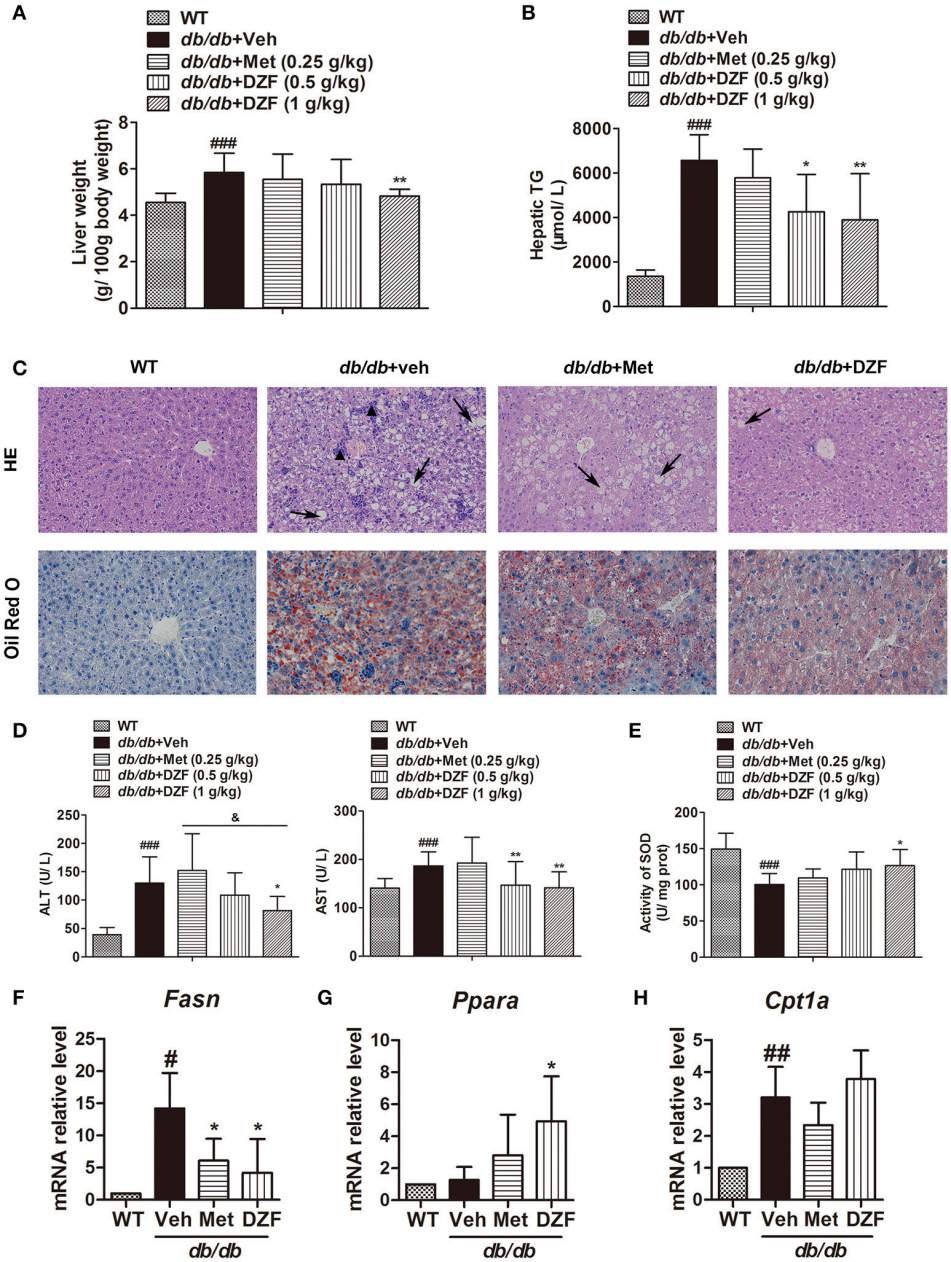
Hepatic steatosis and injury and gene expressions related to lipid metabolism in vehicle-, metformin-, and DZF- (1 g·kg^−1^) treated *db/db* mice. **(A)** Liver weight normalized by total body weight. **(B)** Hepatic TG levels. **(C)** H&E and Oil Red O staining of hepatic tissues. Hepatic steatosis (arrow); inflammatory cells (▴). Original magnification ×200. **(D)** Serum ALT and AST levels. **(E)** SOD activity in the liver of *db/db* mice. **(F–H)** Relative mRNA levels of *Fasn, Ppara*, and *Cpt1a* in the hepatic tissue. Data are described as mean ± SD. **(A,B,D,E)** *n* = 10; **(C)** *n* = 5; **(F–H)** *n* = 3. ^#^*p* < 0.05, ^##^*p* < 0.01 and ^###^*p* < 0.001 vs. WT control; ^*^p < 0.05 and ^**^p < 0.01 vs. *db/db*+ vehicle; ^&^*p* < 0.05 vs. *db/db*+ metformin.

Compared with the wild-type controls, a significant increase in liver weight (g/100 g body weight) was noted in *db/db* mice (*p* < 0.001). DZF (1 g·kg^−1^) lowered liver weight with a significant difference (*p* < 0.01) in comparison with metformin, which exhibited no notable effect (*p* > 0.05, Figure 5A). In *db/db* mice, hepatic TG was much higher than that in wild-type controls (*p* < 0.001, Figure 5B). In this experiment, metformin reduced hepatic TG but with no significant difference (*p* > 0.05). Both doses of DZF remarkably reduced hepatic TG levels (*p* < 0.01). In hematoxylin and eosin (H&E) staining, mixed appearances of microvesicular and macrovesicular steatosis, visible inflammatory cell accumulation, and structural disorganization of hepatocytes were observed in the liver of vehicle *db/db* mice. Hepatic steatosis was more notable in Oil Red O staining as we can observe impressive lipid diffusion (red stain). Such situations were reversed in the DZF groups, wherein lipid droplets decreased in the hepatic tissues. However, the effect of metformin was not as significant (Figure 5C).

#### Hepatic injury

Serum alanine aminotransferase (ALT) and aspartate aminotransferase (AST) activities were determined to evaluate liver functions. Although ALT and AST levels were significantly higher in the vehicle *db/db* mice than that in wild-type controls (*p* < 0.001 and *p* < 0.05, respectively), they notably decreased in DZF-treated mice (*p* < 0.05), especially with higher dose of DZF (1 g·kg^−1^) administration. As depicted in Figure 5D (*p* > 0.05), a notable decrease in hepatic superoxide dismutase (SOD) activity was observed in vehicle *db/db* mice (*p* < 0.001), indicating serious hepatic injury caused by lipid peroxidation. With DZF (1 g·kg^−1^) administration, hepatic SOD activity remarkably increased compared with that in vehicle *db/db* mice (*p* < 0.05). DZF (0.5 g·kg^−1^) and metformin administration raised SOD activity with no significant difference (*p* > 0.05, Figure 5E).

#### Gene expressions of FA synthesis and oxidation in hepatic tissues

To investigate whether alteration of gene expressions related to lipid metabolism occurred in the hepatic tissue, mRNA levels of fatty acid synthase (*Fasn*, for FA synthesis) and peroxisome proliferator-activated receptor alpha (*Ppara*) and carnitine palmitoyltransferase 1a (*Cpt1a*), which are associated with FA oxidation, were detected by quantitative reverse transcription polymerase chain reaction (qRT-PCR). We observed the strong activation of *Fasn* in vehicle *db/db* mice (*p* < 0.05) and a notable reduction in both metformin- and DZF-treated *db/db* mice (*p* < 0.05, Figure 5F). On the other hand, *Ppara*, but not *Cpt1a*, was significantly up-regulated in DZF-treated mice (*p* < 0.05, Figures 5G,H).

### DZF activates AMPK and inhibits notch signaling in the liver of *db/db* mice

**Figure 6:**
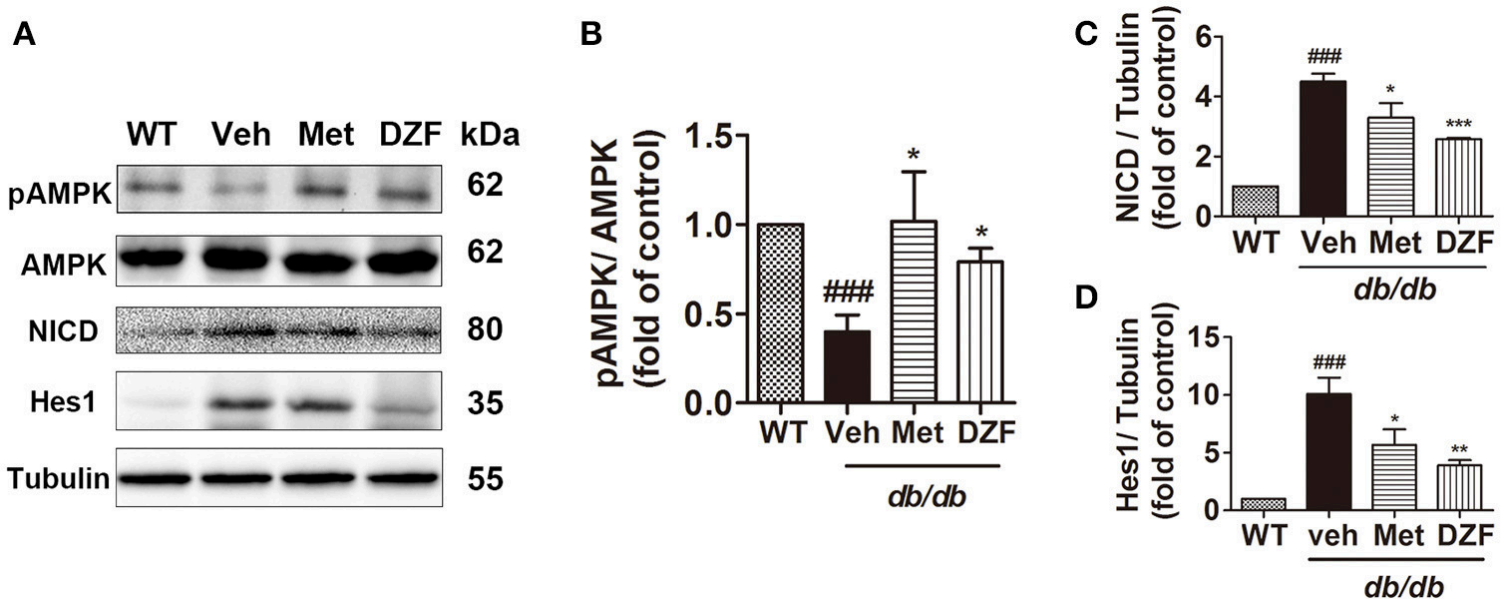
Representative immunoblots and relative expressions of AMPK and Notch signaling proteins in the liver of wild-type and vehicle-, metformin-, and DZF- (1 g·kg^−1^) treated *db/db* mice. **(A)** Representative immunoblots of pAMPK, AMPK, NICD, Hes1, and tubulin in liver. **(B–D)** Relative expressions of pAMPK, NICD, and Hes1 in the liver. Data are described as mean ± SD (*n* = 3). ^###^*p* < 0.001 vs. WT control; ^*^p < 0.05, ^**^p < 0.01 and ^***^p < 0.001 vs. *db/db*+ vehicle.

Western blots presented a significant reduction in AMPK phosphorylation in vehicle *db/db* mice (*p* < 0.001), whereas metformin and DZF increased pAMPK expression compared with that in vehicle *db/db* mice (*p* < 0.05). Two key proteins in the Notch signaling pathway, the Notch intracellular domain (NICD) and its downstream targets Hairy/enhancer-of-split 1 (Hes1), were evaluated. NICD is the activated form of Notch signaling. Results showed a distinct increase in NICD and Hes1 expressions in the vehicle *db/db* mice compared with wild-type controls (*p* < 0.001). However, DZF and metformin significantly reduced NICD and Hes1 expressions compared with those in the vehicle mice (*p* < 0.001, *p* < 0.01, and *p* < 0.05). These results strongly indicate that DZF and metformin can inhibit the Notch signaling pathway in the liver of *db/db* mice (Figure 6).

### Hepatic glycogen content after drug administration in *db/db* mice

**Figure 7:**
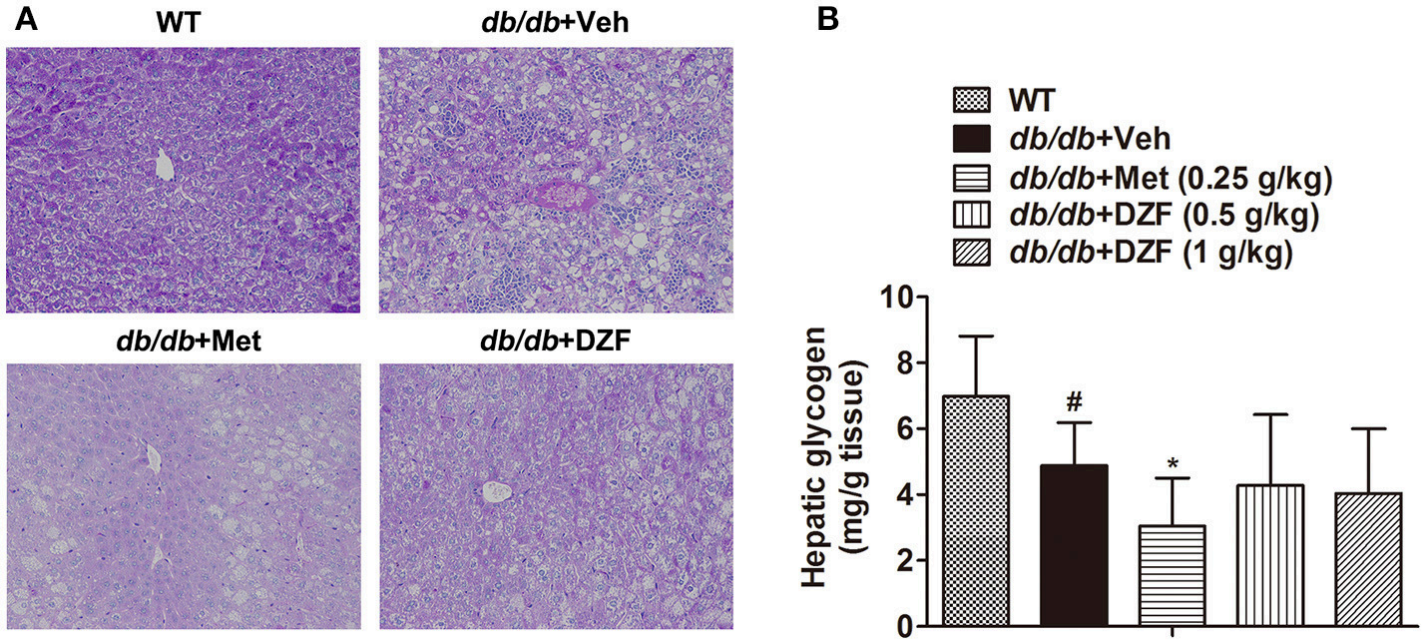
Hepatic glycogen content in wild-type controls and vehicle-, metformin-, and DZF-treated *db/db* mice. **(A)** PAS staining of liver tissues. Original magnification ×200 (*n* = 5). **(B)** Hepatic glycogen content in wild-type and *db/db* mice (*n* = 10). Data are described as mean ± SD. ^#^*p* < 0.05 vs. WT control; ^*^p < 0.05 vs. *db/db*+ vehicle.

Hepatic glycogen content, as an important indicator of glucose metabolism, was assessed by periodic acid Schiff (PAS) staining and assay kit. In PAS staining (Figure 7A), glycogen was stained as red and purple and nucleus blue. The liver sections were deeply stained in wild-type mice, less stained in the vehicle *db/db* mice, and the least stained in metformin-treated mice. No evident glycogen distribution was observed in the DZF-treated mice compared with vehicle *db/db* mice. Glycogen assay kit assessment (Figure 7B) showed the same results with PAS staining. Reduction in glycogen was observed in vehicle *db/db* mice compared with the wild-type controls (*p* < 0.05), whereas metformin caused a more significant reduction and much lower observed values (*p* < 0.05). DZF presented no specific effects on glycogen compared with the vehicle *db/db* mice (*p* > 0.05).

### Effect of drug administration on pancreas of *db/db* mice

**Figure 8:**
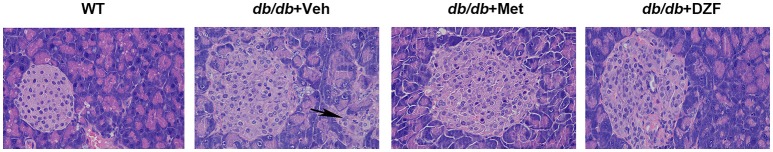
Pancreases from wild-type controls and vehicle-, metformin-, and DZF-treated *db/db* mice. Some islet cells of vehicle *db/db* mice extending to the exocrine portion (arrow). (H&E, original magnification ×400. *n* = 5).

In wild-type control mice, the pink-stained pancreatic islets were well-structured. The borders of these structures were distinctive from the exocrine glands, which were red-stained because of adequate zymogen granules. In vehicle *db/db* mice, we can visibly observed hypertrophic islets, blurred borders, and islet cells extending to the exocrine portion. In metformin- and DZF-treated mice, though the islets remained hypertrophic compared with the wild-type controls, the islet cells were regularly distributed, and the borders were better trimmed than those in vehicle *db/db* mice (Figure 8).

### Effects of DZF on skeletal muscles of *db/db* mice

#### DZF improves ultrastructural alteration of skeletal muscles in *db/db* mice

**Figure 9:**
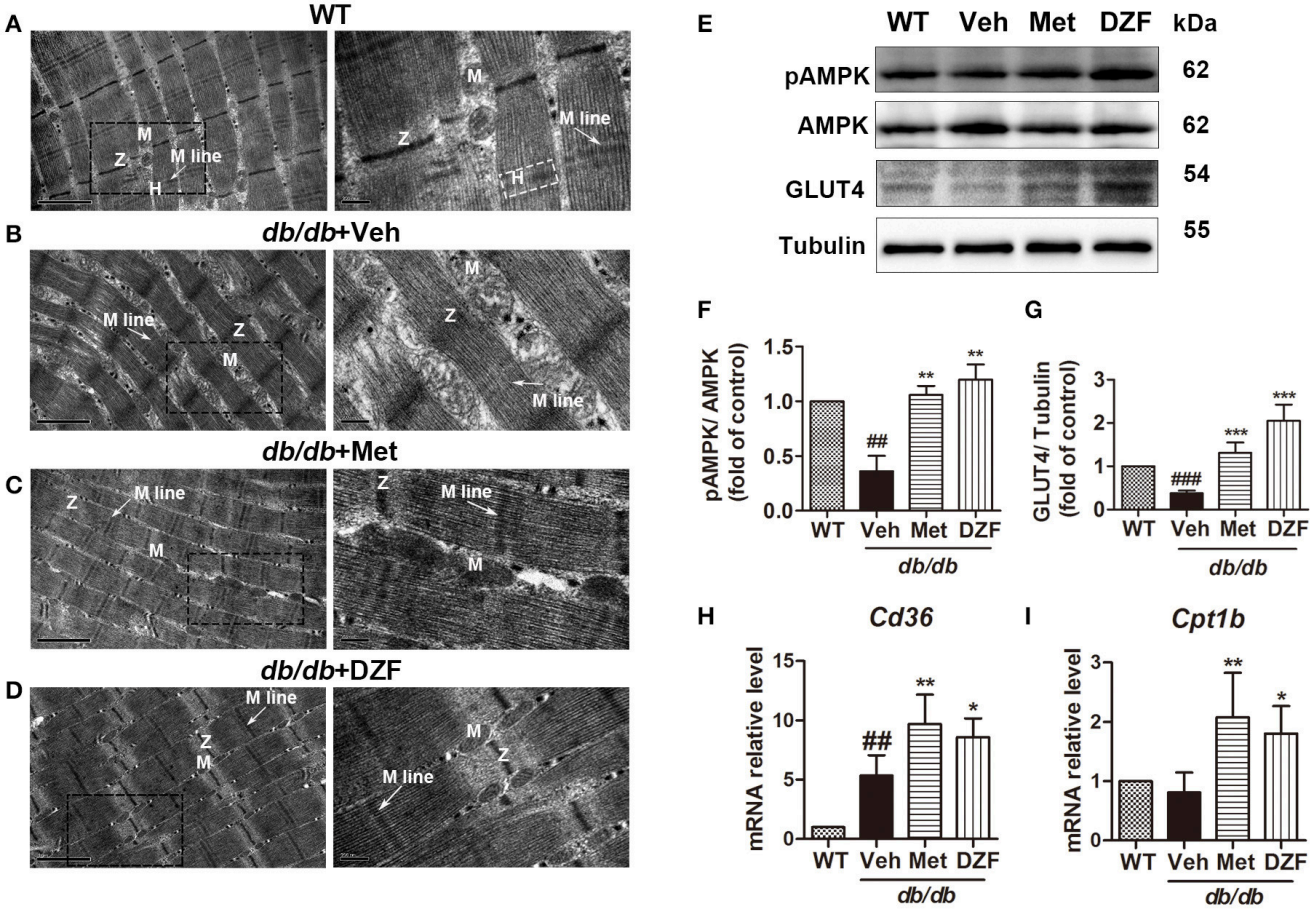
Ultrastructure alteration **(A–D)**, protein **(E–G)**, and gene expressions **(H,I)** of the skeletal muscle in wild-type controls and vehicle-, metformin-, and DZF- (1 g·kg^−1^) treated *db/db* mice. **(A–D)**: TEM; label: Z lines (Z), H zones (H), M lines (arrow), and mitochondria (M). Left panel: scale bar = 1 μm, original magnification ×30000; right panel: scale bar = 200 nm, original magnification ×80000. **(E–G)** Representative immunoblots and relative expressions of pAMPK in muscle and GLUT4 in plasma membrane fraction of the skeletal muscle. **(H,I)**: relative mRNA levels of *Cd36* and *Cpt1b* in the skeletal muscle. Data are described as mean ± SD. **(A–I)** *n* = 3. ^##^*p* < 0.01, and ^###^*p* < 0.001 vs. WT control; ^*^p < 0.05, ^**^p < 0.01, and ^***^p < 0.001 vs. *db/db*+ vehicle.

To observe ultrastructural alteration of skeletal muscles, transmission electron microscopy (TEM) was applied. TEM showed that the skeletal muscles of wild-type control mice presented well-defined myofibrils and sarcomeric patterns with ordered and distinct Z lines, H zones, and M lines. Mitochondria lay between two adjacent myofibrils (Figure 9A). By contrast, the skeletal muscle of vehicle *db/db* mice displayed disarrayed, dissolved, or ruptured myofibrils, thickening and vague Z lines, and blurred H zones and M lines. Especially, the mitochondria appeared enlarged and swollen (Figure 9B). However, the muscle fibers and mitochondrial morphology of metformin- and DZF-treated mice were normalized compared with vehicle *db/db* mice (Figures 9C,D).

#### DZF up-regulates *Cd36* and *Cpt1b* in the skeletal muscles of *db/db* mice

mRNA levels of cluster of differentiation 36 (*Cd36*, for FA transport into cells) (Figure 9H) and *Cpt1b* (for FA transport into the mitochondria and for FA oxidation) (Figure 9I) of skeletal muscles were detected by qRT-PCR. In vehicle *db/db* mice, *Cd36* expression was higher than those of the wild-type controls (*p* < 0.01), and both metformin and DZF can further up-regulate its expression (*p* < 0.01 and *p* < 0.05, respectively). *Cpt1b* also increased with metformin and DZF administration (*p* < 0.01 and *p* < 0.05, respectively), indicating that both compounds can promote FA transport and oxidation in the skeletal muscles of *db/db* mice.

#### DZF activates AMPK phosphorylation and GLUT4 translocation in the skeletal muscles of *db/db* mice

Relative expressions of pAMPK in the skeletal muscle and GLUT4 in the plasma membrane were investigated. Figures 9E–G show a significant reduction in AMPK phosphorylation in the vehicle *db/db* mice (*p* < 0.01) and an increase in both metformin- and DZF-treated mice (*p* < 0.01). GLUT4 expression in the plasma membrane was lower in the vehicle *db/db* mice compared with that in wild-type controls (*p* < 0.001). DZF and metformin treatment can significantly increase GLUT4 expression (*p* < 0.001), indicating the reinforced GLUT4 translocation to the plasma membrane in the skeletal muscles.

## Discussion

Although IR is a general phenomenon of many physiological states, it has long been incriminated in various diseases or disease states (Straub, 2014). In the present study, we observed that TCM herbal formula DZF exerted a satisfactory effect on IR, with improved glucose tolerance, HOMA-IR level, and activated insulin signaling pathway (Figure 3). The effect of DZF on hepatic steatosis was equally notable. Hepatic steatosis, manifested as excessive accumulation of lipids in the liver, refers to lipid formation in lipid droplets of hepatocytes and usually results from lipid synthesis or oxidation perturbation. Hepatic lipids possibly act as markers for the underlying and causative insult for IR (Farese et al., 2012). Hepatic steatosis is a dominant characteristic of NAFLD, which may develop into or lead to cirrhosis, hepatocellular failure, carcinoma, and cardiovascular diseases (Haas et al., 2016). Over 90% of obese and diabetic patients present NAFLD (Tolman et al., 2007). A clinical study showed intrahepatic TG content as the best predictor of progressive impairment of insulin action in the liver, skeletal muscles, and adipose tissues (Korenblat et al., 2008). Our data showed that DZF can significantly reduce hepatic TG and liver weight, with reduction of hepatic lipid accumulation shown in H&E staining and Oil-Red O staining (Figure 5), indicating that DZF can alleviate hepatic steatosis.

Serum NEFA level was also reduced (Figure 4E). We supposed that DZF may inhibit hepatic lipogenesis or promote FA oxidation in both liver and skeletal muscles. Such result was confirmed by qRT-PCR, which showed a reduction in *Fasn* and an increase in *Ppara* mRNA level (Figures 5F,G) in the hepatic tissues and up-regulation of *Cd36* and *Cpt1b* in the skeletal muscles of *db/db* mice (Figures 9H,I). FAs are essential substrates for TG synthesis and is second to glucose as the main fuel for energy production (Samuel and Shulman, 2012). However, excessive FAs contribute to ectopic lipid accumulation and IR. Previous studies showed that glucose utilization improved with chronically increasing FA oxidation in muscle via CPT1 (Bruce et al., 2009); this result coincides with our study, where simultaneous increase in GLUT4 translocation (Figure 9E) and FA transport and oxidation were observed in skeletal muscles. LDL-C is the most important risk factor for cardiovascular diseases, such as myocardial infarction and vascular death (PSC et al., 2007; Ridker, 2014). DZF (1 g·kg^−1^) significantly reduced serum LDL-C (Figure 4B). Reduction of LDL is strongly associated with reduced vascular events (CTTC et al., 2010).

Notch promotes *de novo* lipogenesis possibly through stabilizing mammalian target of rapamycin complex 1 (mTORC1), which can be activated by Akt phosphorylation and promote lipogenesis. However, inhibition of Notch signaling can uncouple Akt from steatosis by decreasing mTORC1 stability (Pajvani et al., 2013). Hes1, one of the main Notch targets, is significantly correlated with plasma insulin level, HOMA-IR, and ALT level (Valenti et al., 2013). Western blots showed activated AMPK and inhibited Notch signaling in the liver (Figure 6) of DZF-treated mice; this result may partly explain how DZF caused reduction in hepatic lipid accumulation and alleviated hepatic injury.

In the present study, no significant reduction was observed in hepatic TG concentration in metformin-administered mice (Figure 5B). This result agrees with those of previous studies, which showed that metformin increased hepatic insulin sensitivity without affecting liver fat in patients with T2DM (Tiikkainen et al., 2004; Teranishi et al., 2007). As metformin has long been assumed to reduce weight in obesity treatment (Marchesini et al., 2001), the present observation of weight-gain tendency contradicted clinical results (Figure 2B). However, such phenomenon is consistent with some previous studies concerning *db/db* mice (Liu et al., 2015; Zheng et al., 2015) or Zucker diabetic fatty rat (ZDF, *fa/fa*) (Wessels et al., 2014). One possible explanation for the effect is that as the *db/db* mice and ZDF rats are leptin receptor-deficient models, any leptin or leptin receptor agonists cannot decrease body weight, but pose a weight-reduction effect on other models (Liu et al., 2015). Metformin can increase leptin receptor expression (Tang et al., 2016), which may be one reason for the weight-loss effect of this compound in other models, but not in the leptin receptor-deficient *db/db* mice. As shown in Figure 3E, no significant difference was observed in serum leptin concentration between the groups of *db/db* mice, indicating the absence of effects on leptin with metformin or DZF treatment in leptin receptor-deficient *db/db* mice. Surprisingly, serum leptin decreased compared with wild-type controls, contradictory to the results of previous studies (Ae Park et al., 2006; Ge et al., 2010). The distinct results may be due to varied observation stages of *db/db* mice. DZF, although reducing hepatic lipids and promoting FA oxidation, caused no changes in body weight (Figure 2B). A previous *in vitro* study showed that DZF promoted lipolysis in 3T3-L1 adipocytes without increasing FA concentration in cell culture supernatant (Zhu et al., 2015). Thus, potential alteration of body composition possible occurred (high lean mass and less fat mass), requiring further studies for verification.

Metformin was previously reported to increase hepatic glycogen (Zheng et al., 2015; Xu et al., 2016). In our studies, however, hepatic glycogen content remarkably decreased according to PAS staining and assay kit assessment (Figure 7). We observed the same result in other studies (Otto et al., 2003; Wang et al., 2012; Xiong et al., 2013). The conflicting results may be worth considering. On the one hand, as an insulin sensitizer, metformin should increase hepatic glycogen by allosteric activation of glycogen synthase (GS). However, on the other hand, metformin is also an AMPK activator, which can acutely inhibit glycogen synthesis by inhibitory phosphorylation of GS (Jeon, 2016). Chronic AMPK activation indirectly increases glycogen synthesis by enhancing glucose uptake and allosteric activation of GS (Hunter et al., 2011; Jeon, 2016). AMPK inhibits lipogenesis and activates lipolysis, whereas insulin signaling promotes lipogenesis. In the present study, we observed that both AMPK and insulin signaling were activated by metformin and DZF administration (Figures 3, 6). However, their effects diverge. DZF caused no significant changes in hepatic glycogen content, different from the result of metformin in *db/db* mice, but reduced hepatic lipids better than metformin (Figure 5B). Correspondingly, DZF reduced serum insulin superior to metformin. (Figure 3C). A balance may exist between insulin signaling action and AMPK effect. The dose administered may be an important factor. Nevertheless, this speculation needs further detection.

The effects of DZF on FBG should not be overlooked. In the first 8 weeks, a significant reduction in FBG was detected after DZF administration, whereas at the end of the experiment, the difference became insignificant (Figure 2A). C57/ BKS- *db/db* mice develop serious diabetes over time (Davis et al., 2010). However, DZF is clinically used in the prediabetic state and metabolic syndrome with characteristic abdominal obesity. Results showed that DZF may not reverse hyperglycemia progression in serious diabetic context. However, DZF remarkably reduced serum insulin concentration. Some arguments claimed that hyperinsulinemia may indicate a primary disruption that drives IR in obesity (Shanik et al., 2008), as the blockade of hyperinsulinemia prevents obesity while increasing energy expenditure (Czech, 2017). Fasting insulin, not glucose nor HbA1c, is associated with coronary artery calcification and its progression (Yamazoe et al., 2016). Although the hypoglycemic effect is not as significant as metformin, DZF improved hyperinsulinemia and general metabolic homeostasis. Such improvement may benefit diabetic prognosis, thus requiring further research.

In this study, in addition to the liver tissue, we observed ultrastructural alteration in skeletal muscles of *db/db* mice. Altered morphology results in biochemical dysfunction of mitochondria (Vogel, 2001). Through TEM, we observed the normalized shape, number, and distribution of mitochondria in the myocytes of DZF-treated mice (Figures 9A–D). Western blots showed increased translocation of GLUT4 to the plasma membrane and activation of AMPK phosphorylation in DZF-treated mice (Figure 9E), indicating that DZF may increase glucose uptake and promote energy metabolism in the skeletal muscles of diabetic mice. However, we need further investigation in the future to determine the exact effects of DZF on skeletal muscle mitochondria and fuel metabolism with quantitative determination.

In conclusion, DZF, as a TCM herbal formula containing berberine, naringin, and other components, exhibits a prominent effect in improving insulin sensitivity, hepatic steatosis, and skeletal muscle energy metabolism in *db/db* mice. DZF activated AMPK and FA oxidation in liver and skeletal muscle tissues, inhibited Notch signaling in liver, and promoted GLUT4 translocation in skeletal muscles. As a main component of DZF, berberine has the hypoglycemic, hypolipidemic, and insulin sensitizing effect (Yin et al., 2008; Zhang et al., 2008; Wang et al., 2011; Pirillo and Catapano, 2015), attenuating hepatic steatosis (Sun et al., 2018), and activates AMPK and GLUT4 in the skeletal muscle (Lee et al., 2006). Naringin, in addition to its potent antioxidant nature, affects AMPK-, CPT1–, and PPARa– mediated fat utilization, and also preserves mitochondrial function in the treatment of diabetes, metabolic syndrome, and associated complications (Alam et al., 2014). To the best of our knowledge, we observed no defined berberine and naringin effects on Notch signaling in liver and the coexistence of all effects illustrated in this study. All these effects that DZF achieved are not likely to depend on a particular component. Berberine may primarily contribute to DZF effects, and whether DZF effects are superior to berberine necessitates further investigation.

**Figure 10:**
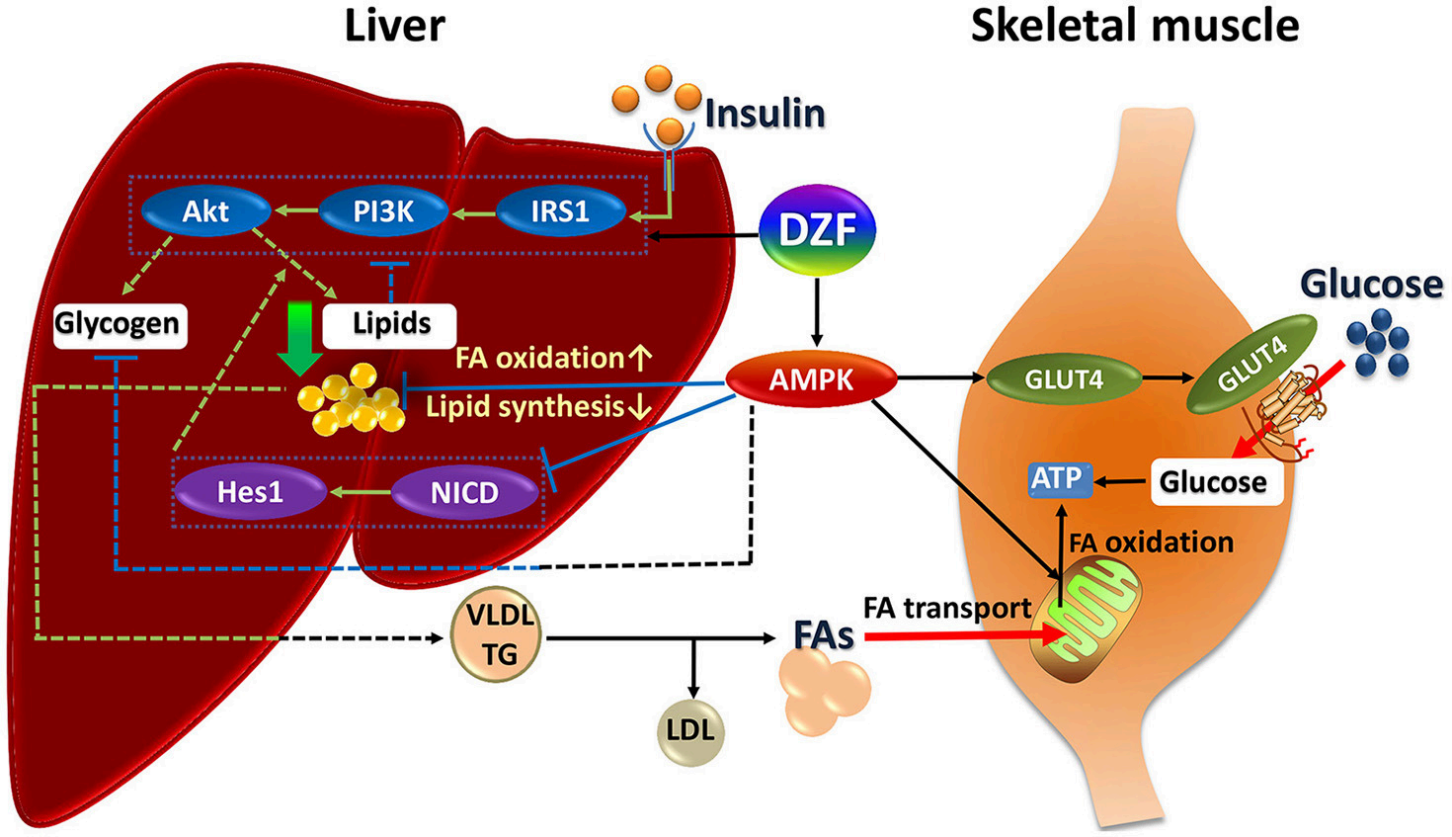
Summary of DZF effects on liver and skeletal muscle in insulin-resistant and diabetic *db/db* mice. Dashed lines: processes in the present study that were suppressed or indistinct with DZF administration partly due to interactions of different signaling effects. Dotted square: key proteins of insulin signaling and Notch signaling. FA, fatty acid; VLDL, very low– density lipoprotein; TG, triglyceride.

The mechanism of DZF defies explanation by a single pathway, but correlates with the effect of multiple interactions. The underlying mechanism of DZF in *db/db* mice may be as follows (Figure 10). DZF activates AMPK in the liver, reducing hepatic lipids by inhibiting lipogenesis and promoting FA oxidation. Inhibition of Notch signaling pathway by AMPK activation uncouples Akt from steatosis. Hepatic lipids can be causative of IR, and as a result, IRS-1/PI3K/Akt signaling will be inhibited. With hepatic lipid reduction, DZF indirectly activates hepatic insulin signaling pathway, improves hyperinsulinemia, and promotes glycogen synthesis. However, the glycogen-associated effect can be balanced with inhibition from AMPK. The hepatic tissue secretes TG-rich lipoproteins (i.e., very-low-density lipoprotein-triglyceride, VLDL-TG) into circulation, which then turns into FAs with the effect of lipoprotein lipase (Goldberg and Ginsberg, 2006). FAs are transported to the mitochondria of skeletal muscles. The well-structured mitochondria provide a fine platform for FA β-oxidation, which may generate considerable ATP. On the other hand, DZF activates AMPK in the skeletal muscles and promotes GLUT4 translocation to the plasma membrane, thus increasing glucose uptake, which proves critical in decreasing blood glucose and generating ATP in cells. The whole process has not been fully verified, but we hope that such a model may provide some ideas for treatment of IR from a systemic point of view.

## Materials and methods

### HPLC analysis

Samples of DZF extract were separated on an Agilent Zorbax SB C18 column (4.6 × 250 mm^2^, 5 μm, USA), and mobile phases consisted of solvents A (pure water) and B (acetonitrile). A gradient eluting program was selected as follows: 0–14 min, 78% A with 22% B; 14–15 min, linear gradient elution 22−30% B; 15–25 min maintaining 30% B for 10 min; 25–28 min, linear gradient 30−60% B; maintaining 60% B for 10 min. Flow rate was 1.0 ml/min, and volume for sample analysis was 10 μL injection. Reference substances of berberine, palmatine, jatrorrhizine, naringin, hesperidin, and neohesperidin were purchased from the National Institutes for Food and Drug Control (Beijing, China).

### Animals

All experimental procedures in the study were in compliance and approved by the Ethics Committee of the Institute of Medicinal Plant Development, CAMS & PUMC (Beijing, China) and were carried out according to the Guide for the Care and Use of Laboratory Animals published by the National Institutes of Health (NIH Publications No. 85-23, revised 1996). A total of 40 male *db/db* mice and 10 male wild-type C57BL/Ksj mice were purchased from Nanjing Biomedical Research Institute of Nanjing University (Nanjing, China) at 6 weeks of age. Animals were housed at 25 ± 1°C, 55−65% relative humidity, and a light/dark cycle of 12 h. All mice were given free access to food (standard chow diet) and drinking water. Best efforts were exerted to minimize mice suffering. After 2 weeks of acclimation, *db/db* mice were randomly divided into 4 groups (*n* = 10) and were treated daily via gavage with vehicle (distilled water, 5 mL·kg^−1^), metformin (BMS, Shanghai, 0.25 g·kg^−1^) and DZF (0.5 g·kg^−1^, 1 g·kg^−1^). Ten wild-type mice, as normal control, were treated with vehicle. After 12 weeks of administration, mice were starved for 12 h and sacrificed under anesthesia by chloral hydrate. Blood samples were collected for serum assessment. Liver, skeletal muscle, and pancreatic tissues were removed and frozen at −80°C until analyzed or fixed in paraformaldehyde (PFA). Livers were weighed for analysis.

### OGTT

On the eleventh week, OGTT was conducted over 12 h of food removal. After baseline blood glucose (0 min) had been measured, mice were given 2 g/ kg of glucose, followed by blood glucose measurement at 30, 60, 90, and 120 min. AUCs were assessed. Blood glucose was measured by an automatic glucometer (One Touch Ultra, Lifescan, USA).

### Serum insulin and HOMA-IR

After 12 weeks of administration, blood samples were collected after a 12 h of overnight starvation and were centrifuged at 3500 rpm for 15 min. Serum insulin was measured using the Mouse Ultrasensitive Insulin Elisa kit (Alpco, USA) according to manufacturer's instructions. Insulin sensitivity was also determined by calculating HOMA-IR according to the following formula (Matthews et al., 1985): HOMA-IR = [fasting glucose (mmol/l)] × [fasting insulin (μU/ml)] /22.5.

### Serum and liver measurements

Serum TG, CHO, HDL, LDL, ALT, and AST levels were assessed by an automatic biochemical analyzer in Guang'anmen Hospital. Serum NEFA, hepatic SOD, and hepatic glycogen contents were measured by assay kits (Nanjing Jiancheng Bioengineering Institute, China). Serum leptin was detected by Mouse Leptin ELISA Kit (Cusabio, China). Hepatic TG was assayed using a TG assay kit (Applygen, China). All procedures were conducted according to manufacturer's recommended protocols.

### Histological analysis

#### H&E staining

Excised liver and pancreas tissues were fixed in 4% PFA, embedded in paraffin, and cut into 5 μm-thick sections. The sections were then stained with H&E and assessed on a light microscope (Nikon Eclipse E100 microscope; Nikon, Tokyo, Japan).

#### Oil red O staining

To further detect hepatic lipid distribution, Oil Red O staining was performed as previously described (Araújo et al., 2016). Briefly, the fixed liver samples (5 per group) were embedded in the presence of liquid nitrogen. Frozen cuts were made on a cryostat (Cryostar NX50, Thermo, USA), and the cuts were fixed in fixation solution for 15 min and stained with Oil Red O for 8–10 min, after which they were rinsed with distilled water. The samples were immersed in 75% ethanol and rinsed again. Then, the nuclei lightly stained with hematoxylin were observed. Images of the same magnification (200×) were collected.

#### PAS staining

To observe hepatic glycogen, PAS staining was performed. First, sections were deparaffinized and hydrated with water and then oxidized in periodic acid solution for 15 min. After rinsing the sections with distilled water, they were placed in Schiff reagent for 30 min and rinsed with tap water for 5 min. Then, the samples were counterstained in hematoxylin for 3 min and rinsed.

### Electron microscopic observations of the skeletal muscle

Gastrocnemius skeletal muscles were cut into 1 mm cubes and were immediately fixed in 2.5% glutaraldehyde. After rinsing thoroughly with phosphate buffered solution, the skeletal muscles were fixed in 1% osmium tetroxide for 2 h, rinsed again, and dehydrated by a graded series of ethanol (30, 50, 70, 80, 90, 95, and 100%) and transferred to absolute acetone for 15 min. Then, the specimens were infiltrated, embedded, and polymerized. After ultrathin sectioning by Leica EM-UC 6, the specimen sections were stained with uranyl acetate and alkaline lead citrate and observed under a transmission electron microscope (HITACHI H-7500).

### Western blots

Tissue fractions from the wild type and vehicle, metformin-, and DZF- (1 g·kg^−1^) treated *db/db* mice were weighed and accordingly added with RIPA and PMSF (Beyotime, China) mix (99:1) at the ratio of 1:10. Lysis was initiated in a high-flux tissue grinding mill. After centrifugation at 13,000 rpm for 20 min, the supernatant was transferred to new centrifuge tubes (to avoid the superficial layer of lipids). Membrane protein extraction of skeletal muscles was conducted according to manufacturer's protocol (BestBio, China). Bicinchoninic acid (BCA) method was used to determine protein concentration. The following Western blotting procedures were carried out as previously described (Chen et al., 2017). Proteins were separated on 10 or 8% SDS-PAGE gels and then transferred onto nitrocellulose membranes. After the membranes were blocked with 5% non-fat milk blocking buffer for 2 h at room temperature, the following primary antibodies were used for overnight incubation at 4°C. Antibodies against IRS1 (2382 S), pIRS1 (Ser 307, 2381 S), pAkt (4060 S), and pAMPK (# 2535) were obtained from Cell Signaling Technology and used at a dilution of 1:1,000; antibodies against α-tubulin (ab 176560), Akt (ab 64148), AMPK (ab 80039), GLUT4 (ab 166704), NICD (ab 8925), and Hes1 (ab 70576) were obtained from Abcam and were used at a dilution of 1:1,000. Then, the membranes were washed thrice with TBST and incubated with secondary antibodies (Zsbio, China) for 1.5 h at room temperature, after which they were washed again as before. Protein bands were visualized after development using an enhanced chemiluminescence solution for 5 min. Western blots were quantified using Gel-Pro Analyzer 4.

### qRT-PCR

Total RNA was extracted with Trizol Reagent from frozen liver and skeletal muscle tissues, and RNA concentrations were measured by a spectrophotometer (Nanodrop 2000c, Thermo Fisher). cDNA synthesis was performed with PrimeScript RT reagent Kit (Takara, Japan). qRT-PCR using SYBR Premix ExTaq reagent Kit (Takara, Japan) was run in the CFX96 Real-Time PCR Detection System (Bio-Rad Laboratories, USA). Primers used were as follows: mouse β*-Actin*: Forward 5′-GGCTGTATTCCCCTCCATCG-3′, Reverse 5′-CCAGTTGGTAACAATGCCATGT-3′; *Cpt1*α: Forward 5′-CTCCGCCTGAGCCATGAAG-3′, Reverse 5′-CACCAGTGATGATGCCATTCT-3′;

*Cpt1b*: Forward 5′-GCACACCAGGCAGTAGCTTT-3′, Reverse 5′- CAGGAGTTGATTCCAGACAGGTA-3′; *Fasn*: Forward 5′-GGAGGTGGTGATAGCCGGTAT-3′, Reverse 5′-TGGGTAATCCATAGAGCCCAG-3′; *Ppara*: Forward 5′-AGAGCCCCATCTGTCCTCTC-3′, Reverse 5′-ACTGGTAGTCTGCAAAACCAAA-3′; *Cd36*: Forward 5′-ATGGGCTGTGATCGGAACTG-3′, Reverse 5′-GTCTTCCCAATAAGCATGTCTCC-3′. The mRNA expressions were calculated according to a comparative method (2^−ΔΔ*Ct*^) using β*-Actin* as control.

### Statistical analysis

Data were expressed as mean ± standard deviation (SD). One-way ANOVA, followed by Newman–Keuls *post hoc* test, was used to compare differences among all groups by Prism 5.0 software (GraphPad Software, La Jolla, CA, USA). *p* < 0.05 was considered statistically significant. All data are the result of at least three replicates.

## Author contributions

XL and XS: designed and supervised the study; LZ: performed the research, analyzed the data and wrote the manuscript; XZ, GS, and XM: contributed to handling the research process and data interpretation; MW: provided extensive revision of the manuscript; HC and JW: contributed to the quality control of DZF extract and revised the manuscript; YZ, KY, and YT: participated in the performance of the experiment and contributed to figure preparation. All authors reviewed and approved the submission of the manuscript.

### Conflict of interest statement

The authors declare that the research was conducted in the absence of any commercial or financial relationships that could be construed as a potential conflict of interest.
